# Malaria infection and disease in an area with pyrethroid-resistant vectors in southern Benin

**DOI:** 10.1186/1475-2875-9-380

**Published:** 2010-12-31

**Authors:** Georgia B Damien, Armel Djènontin, Christophe Rogier, Vincent Corbel, Sahabi B Bangana, Fabrice Chandre, Martin Akogbéto, Dorothée Kindé-Gazard, Achille Massougbodji, Marie-Claire Henry

**Affiliations:** 1 Centre de Recherche Entomologique de Cotonou (CREC), 06 BP 2604 Cotonou, Bénin; 2 Institut de Recherche pour le Développement (IRD/UR016), 01 BP 4414 Cotonou, Bénin; 3 Unité de recherche en biologie et épidémiologie parasitaires, Equipe « Maladies émergentes et moustiques »/Unité de recherche sur les maladies infectieuses et tropicales émergentes, URMITE-UMR6236, Institut de recherche biomédicale des armées, Allée du médecin colonel Jamot, Parc du Pharo, BP60109 Marseille cedex 07, France; 4 Laboratoire de lutte contre les Insectes Nuisibles (LIN/IRD), 911 Ave Agropolis BP 64501, 34394 Montpellier Cedex 5 France; 5 Faculté des Sciences de la Santé/Université d'Abomey-Calavi (FSS/UAC), 01 BP 188 Cotonou, Bénin; 6 Service de Coopération, Ambassade de France, Cotonou, Bénin

## Abstract

**Background:**

This study aimed to investigate baseline data on malaria before the evaluation of new vector control strategies in an area of pyrethroid-resistance of vectors. The burden of malaria was estimated in terms of infection (prevalence and parasite density) and of clinical episodes.

**Methods:**

Between December 2007 and December 2008 in the health district of Ouidah - Kpomassè - Tori Bossito (southern Benin), a descriptive epidemiological survey of malaria was conducted. From 28 selected villages, seven were randomized from which a total of 440 children aged 0 to 5 years were randomly selected. Clinical and parasitological information was obtained by active case detection of malaria episodes carried out during eight periods of six consecutive days scheduled at six weekly intervals and by cross-sectional surveys of asymptomatic infection. Entomological information was also collected. The ownership, the use and the correct use of long-lasting insecticide-treated nets (LLINs) were checked over weekly-survey by unannounced visits at home in the late evening.

**Results:**

Mean parasite density in asymptomatic children was 586 *P. falciparum *asexual forms per μL of blood (95%CI 504-680). Pyrogenic parasite cut-off was estimated 2,000 *P. falciparum *asexual blood forms per μL. The clinical incidence of malaria was 1.5 episodes per child per year (95%CI 1.2-1.9). Parasitological and clinical variables did not vary with season. *Anopheles gambiae s.l*. was the principal vector closely followed by *Anopheles funestus*. Entomological inoculation rate was 5.3 (95%CI 1.1-25.9) infective bites per human per year. Frequency of the L1014F *kdr *(West) allele was around 50%. Annual prevalence rate of *Plasmodium falciparum *asymptomatic infection was 21.8% (95%CI 19.1-24.4) and increased according to age. Mean rates of ownership and use of LLINs were 92% and 70% respectively. The only correct use of LLINs (63%) conferred 26% individual protection against only infection (OR = 0.74 (95%IC 0.62-0.87), p = 0.005).

**Conclusion:**

The health district of Ouidah-Kpomassè-Tori Bossito is a mesoendemic area with a moderate level of pyrethroid-resistance of vectors. The used LLINs rate was high and only the correct use of LLINs was found to reduce malaria infection without influencing malaria morbidity.

## Background

Despite considerable worldwide efforts made in recent years to control malaria [1], the disease is still a major public health problem with nearly 250 million cases and about one million deaths each year. Eighty five percent of deaths occur among children under five [2] from which nearly all are in sub-Saharan Africa. In 2007, malaria was declared to be the most important disease in this age group, in Benin, leading to 43% of all medical consultations and 29% of hospital admissions [3]. The National Malaria Control Programme (NMCP) has implemented WHO/GMP's (World Health Organization/Global Malaria Programme) recommended preventive and curative strategies [4]. These include i) Artemisinin combination therapy (ACT) which is dispensed at health centers and has recently been made available to communities for children under five years old; ii) Intermittent preventive treatment (IPT) during pregnancy; iii) Long-lasting insecticide-treated mosquito nets (LLINs) which have continued to be distributed following the nation-wide deployment among high-risk populations (i.e. children of under five and pregnant women) and iv) Indoor residual spraying (IRS) using carbamate insecticide applied in specific districts through the President's Malaria Initiative [5]. Many studies have demonstrated that the use of insecticide treated nets reduced uncomplicated malaria episodes by at least 50% [6]. Unfortunately, insecticide resistance in malaria vectors has dramatically increased in Africa [7], especially in Benin [8-10] and may seriously compromise the success of vector control management. Two studies conducted in experimental huts in South Benin, where *Anopheles gambiae *was resistant to pyrethroids, have reported that significant reduction in the efficacy of pyrethroids was applied either in treated nets or IRS [11,12]. In order to manage insecticide resistance, the Centre de Recherche Entomologique de Cotonou (CREC) in collaboration with the Institut de Recherche pour le Développement (IRD) and the NMCP has evaluated successfully (WHOPES phases I and II) a new insecticide resistance management (IRM) strategy combining in the same household a LLIN and a carbamate treated plastic sheeting [13,14]. In the context of a future community-based evaluation of this promising IRM strategy (phase III trial), the malaria burden was evaluated in a health district of southern Benin where a nation-wide distribution of LLINs to children <5 had been implemented in 2007. This study constitutes an analysis of the baseline situation of malaria in terms of infection (prevalence and parasite density) and clinical episodes. Entomological information was also collected.

## Methods

### Site description

**Figure 1:**
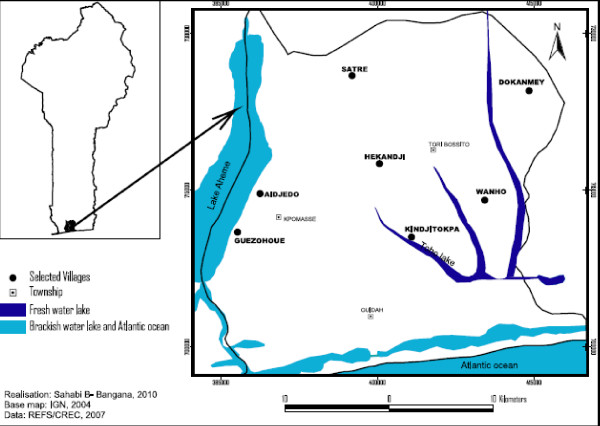
**Map of Ouidah-Kpomassè-Tori Bossito health district, south of Benin, showing the selected villages**.

This epidemiological study was carried out in the Ouidah-Kpomassè-Tori Bossito health district in southern Benin (Figure 1), from December 2007 to November 2008. The population size in the study area was 178,314 inhabitants according to the results of the 3^rd ^General Census of the Population and the Environment (RGPH3) of February 2002. The population is rural and lives on agriculture with scattered settlement. The main ethnic group is Aïzo. The climate is essentially sub-equatorial, with two dry seasons (a long dry season from December to March and a short dry season in August and September), and two rainy seasons (a long rainy season from April to July and a short rainy season from October to November). The average annual rainfall is around 1,200 mm, of which 700-800 mm and 400-500 mm rain down respectively in the first and in the second rainy season. The hottest months (31°C) are February to April and the coldest months (27°C) are July to September. Less than 30% of children are present at the health centre when sick. They are mostly treated by traditional medication [15]. A recent survey in Benin indicated that less than half of febrile children <5 were received anti-malarial drugs of which only 7% of cases were given ACT [16,17]. Malaria vectors show resistance to pyrethroids in south of Benin [18].

### Sampling

Twenty eight villages were chosen according to the following criteria: having between 250-500 inhabitants, distance between any villages greater than two kilometers and the absence of a local health centre. From these, seven villages were randomly selected. Geographical, demographical and environmental characteristics are described in Table 1. After census, about 60 children aged 0-71 months were randomly selected in each village. They were clinically monitored for a total of 48 days spread over one year. Children born during the study were not included. Ethical clearance was given for the study by the National Ethical Committee in Benin (Comité National Provisoire d'Ethique pour la Recherche en Santé, Reference number IRB00006860) and IRD ethical committee. Mosquito collectors gave their written informed consent and were treated free of charge for malaria presumed illness. They were also vaccinated against yellow fever. Each head of family or the guardian of the selected child gave their written informed consent. During the monitoring periods, all children of villages, whom participating in the study or not to, were treated free of charge by the medical staff.

**Table 1:** Description of the study area

Village	Spatial coordinates		Density of population (People/Km^2^)	Environnemental characteristics				
			
	Longitude (°C)	Latitude (°C)		Open water cisterns (N)	Swamps*	Distance from lake (fresh water) (Km)	Distance from lagoon (brackish water) (Km)	Distance from health center (Km)
Aïdjèdo	06° 24'	02° 20'	47	2	-	7.9	0.6	3
Dokanmè	06° 33'	02° 13'	61	5	-	0.5	22.6	4
Kindjitokpa	06° 25'	01° 58'	78	0	+	0.1	9.7	2
Guézohoué	06° 29'	02° 05'	91	0	-	11.1	0.5	2
Hékandji	06° 25'	02° 07'	67	4	-	4.0	10.0	5
Satré	06° 34'	02° 04'	45	17	-	13.8	8.5	4
Wanho	06° 27'	02° 11'	63	0	+	2.2	12.2	2

* Presence of swamps (+) and absence of swamps (-)

### Data collection

#### Parasitological and clinical measures

Active case detection (ACD) for malaria episodes was carried out during eight periods of six consecutive days at six weeks intervals throughout the year. Each day a nurse assisted by a local village helper trained for the study, visited the households in the sample. A physician supervised the field work. The presence or absence and state of health of each child were recorded daily on a specially prepared form (one form per household). The nurse examined and recorded data on every case of sickness detected at home. A thick blood film was taken from every sick child. Children were treated according to the clinical diagnosis made by the nurse. When malaria was suspected, the patient was treated with artemether-lumefantrine for three days according to the recommendations of WHO and NMCP [19,20]. Cross-sectional surveys (CSS) were carried out at each monitoring clinical period (n = 8) on every asymptomatic child (confirmed by axillary temperature < 37.5°C). A thick film sample was taken on the fourth day to be sure that asymptomatic children were free of illness in preceding days. Cross-check quality controls were conducted every six weeks during the collection of field data.

#### Entomological measurements

Data were collected two weeks before each clinical monitoring. Adult mosquitoes were caught using Human Landing Catches (HLC) technique [21]. In the study area, 896 human-nights of capture of human landing mosquitoes were organized every six weeks over a year period (128 nights per village; eight places per village and per night, half indoor and half outdoor). Treated nets were present in the mosquito collection sites. The mosquito species were identified using morphological characteristics according to the identification keys of Gillies & De Meillon [22] and Gillies & Coetzee [23]. All mosquitoes of *An. gambiae *complex and *Anopheles funestus *group were stored in individual tubes with silicagel and preserved at -20°C for *P. falciparum *circumsporozoite index estimation and molecular identification.

#### Control of LLINs

The ownership, the use and the correct use of LLINs (Permanet^® ^2.0) which were distributed in October 2007 were checked over weekly-survey. The visits of the nurse were unannounced and took place in the late evening around 9.00 PM when children were expected to be asleep [24]. The unannounced visits determined the ownership (whether the LLINs were seen during the control), the use (whether children were sleeping under it during the control) and the correct use (whether the LLINs were correctly hung and tucked and were not torn). The rates of LLINs ownership, use and correct use were calculated relative to the total number of observations.

#### Laboratory examination

Laboratory processing was done at the CREC, Cotonou. Parasitological infection was detected on Giemsa-stained thick smears. Asexual stages of each *Plasmodium *species were counted in the blood volume occupied by 200 leucocytes and parasite density was calculated by assuming 8,000 leucocytes/μL of blood. Thick smears from each village were read by the same experienced technician, under the supervision of a parasitologist. The readings of the two technicians were also compared on the same set of blood samples. Their estimations of parasite detection and parasite density did not differ significantly. Cross-check quality control was regularly done on a randomly selected sample representing 10% of all thick smears.

After scoring field-collected Anopheles mosquitoes and identifying the species of each specimen by Polymerase Chain Reaction (PCR) [25], the presence and relative frequency of the molecular M and S forms of *An. gambiae sensu stricto *(s.s) were determined according to the method of Favia [26]. Infection of mosquitoes was determined on the head and thorax of individual vector specimens by ELISA using monoclonal antibodies against *P. falciparum *circumsporozoite protein (CSP) [27]. The method of Martinez-Torrez was used for the molecular detection of the L1014F *kdr *allele [28].

#### Data analysis

Demographic, parasitological, clinical and entomological data were double entered independently in the Access 2003 database. Parasitological and clinical data were analyzed using the svy command (STATA 11.0). For each person only one blood sample per monitoring period was considered for analysis. When a pathological condition was detected, the blood sample taken during the clinical episode was retained for analysis. Parasitological data were analyzed separately in terms of prevalence of *P. falciparum *asexual blood forms, density of *P. falciparum *asexual blood forms in parasite positive blood thick films and prevalence of *P. falciparum *gametocytes. A generalized estimating equation (GEE) approach, which can be used with normal distributions and discrete data was used for statistical analysis of repeated measures. To take into account the interdependence of observations made on the same person, an exchangeable correlation structure was used in which the correlation between these observations made on one person at different times was assumed to be the same. The prevalence of asymptomatic malaria infections was analyzed as a binomial response by using a logistic regression model. The parasite density was log transformed for a normally distributed response and analyzed with a link function by using a linear regression model.

The association between the parasite density and the occurrence of clinical episodes was tested using a Poisson regression model, taking clinical status (pathological episode *versus *asymptomatic state) as the dependent variable, and parasite density as the independent variable. In this type of model, a random intercept variable is allowed to vary with subjects, and this random subject-specific intercept allows the interdependence of the observations made on the same person to be taken into account. For each pathological period, the probability that it was caused by malaria was estimated by the Attributable Fraction (AF) calculated from the odds ratios associated with the estimated parasite density in the logistic model [29,30]. The pathological episodes were clinically defined by a high axillary temperature (≥ 37.5°C), sweats, shivers, headaches, nausea or vomiting [31] or by a history of fever during the 48 hours proceeding the first day of ACD or, for infants under one year of age, anorexia or any pathological condition described by the mother [32,33]. For individuals, the number of malaria attacks over a given periods was estimated by the sum of probabilities that pathological episodes were due to malaria, depending on the parasite density. The malaria incidence rate was calculated dividing the ratio of pathological episodes attributable to malaria by the number of child-days.

The three dependent variables (i.e. prevalence rate of *P. falciparum *infection, mean parasite density in positive children and clinical incidence rate) were analyzed according to demographic (age groups 0-23, 24-59, 60-71 months and sex), environmental (season and villages) and sanitary (LLIN's ownership, use and good use) variables. The Chi^2 ^test was used to compare the rate of ownership, use and correct use of LLINs. An optimum pyrogenic parasite density cut-off was calculated using the estimated AFs with a logistic model. The sensitivity and the specificity were similarly determined [34]. The sensitivity was estimated by the ratio of malaria episodes with positive cut-off to a total of malaria episodes. The specificity was estimated by the ratio of no malaria febrile episodes with parasite density below the cut-off to the total of no malaria febrile episodes. The suitable positive Likelihood-ratio (>10), negative Likelihood-ratio (<0.1) results and Youden's J index were also determined from the model.

The human biting rate (HBR) was expressed as the number of anopheles bites per human per night. The sporozoite index was calculated as the proportion of mosquitoes found to be positive for CSP. The entomological inoculation rate (EIR) was calculated as the product of the HBR and the sporozoite index and expressed as the number of infected bites per human per year.

## Results

### Population description

A total of 440 children in seven villages were parasitologically and clinically monitored during 18,262 person-days from which 402 (2.2%) were missing for the following reasons: 366 not found and 36 refusals. Ten children died during the study. The mean age of the children at inclusion was 2.1 years. The female/male ratio was 1:1. Each child in the survey was visited on an average of 42 days out of the 48. A total of 3,074 thick blood films were taken, comprising 2,838 in asymptomatic children and 236 in sick children, with an average of seven per child.

### Parasitological indexes of asymptomatic children observed by CCS

*Plasmodium falciparum, Plasmodium malariae *and *Plasmodium ovale *were present alone or mixed (Table 2). The annual prevalence rate of *P. falciparum *infection was 21.8% (95%CI 19.1-24.4). In the multivariate random-effects logistic regression model, age of children, season, village and correct use of LLINs, but not the ownership and the use of LLINs were significantly associated with the prevalence of infection (Table 3). The correct use of LLINs conferred a 26% individual protective effect against infection prevalence (OR = 0.74 (95%CI 0.62-0.87), p = 0.005). The prevalence of infection increased with age. Children aged 1 to 2 years and 3 to 5 years were three to five times more frequently infected than children aged less than one year (22.0% (CI95% 17.0-27.0) and 33.0% (CI95% 28.4-37.6) *versus *7.8% (CI95% 5.2-10.5)). The prevalence of infection was higher during the dry season (24.7% (CI95% 21.6-27.8) than during the rainy season (18.6% (CI95% 15.7-21.5)). The prevalence of infection was higher in Satré, Wanho, Kindjitopka and Hèkandji than in Dokanmè, Aidjèdo and Guézohoué.

**Table 2:** Distribution of Plasmodium species according to clinical status.

*Plasmodium *species	Asymptomatic children	Sick children	Total
*Pf*	593	102	695
*Pf+Pm*	9	0	9
*Pf+Po*	15	3	18
*Pf+Pm+Po*	1	0	1
*Pm*	21	3	24
*Pm+Po*	1	0	1
*Po*	14	2	16

Total	654	110	764

*Pf*: *Plasmodium falciparum*; *Po*: *Plasmodium ovale*; *Pm*: *Plasmodium malariae*. The plamodial formula on the 764 of the *Plasmodium*-positive thick blood film is *Pf *= 0.95; *Pm *= 0.05; *Po *= 0.05.

**Table 3:** Multivariate analysis of the prevalence rates of Plasmodium falciparum asymptomatic infection determined by cross-sectional surveys.

	Prevalence rate of *P. falciparum *asymptomatic infection				
	
Variable	N positive/N total (%)	95%CI	OR	95%CI	p-values
**Age (years)**					
<1	67/857(7.8)	5.1-10.6	1		
1-2	205/932(22.0)	16.7-27.3	3.62	2.52-5.19	<0.001
3-5	346/1049(33.0)	27.9-38.0	5.15	3.67-7.23	<0.001
**Village**					
Aïdjèdo	59/427(13.8)	8.8-18.8	1		
Dokanmè	77/414(18.6)	12.8-24.4	1.55	0.89-2.72	0.103
Kindjitokpa	99/405(24.4)	20.8-28.1	2.12	1.34-3.36	0.007
Guézohoué	27/410(6.6)	4.2-8.9	0.48	0.28-0.84	0.019
Hékandji	82/392(20.9)	15.8-26.0	1.68	1.00-2.81	0.049
Satré	129/364(35.4)	30.7-40.2	3.42	2.16-5.43	0.001
Wanho	145/426(34.0)	21.1-39.0	3.33	2.09-5.31	0.001
**Season**					
Dry	366/1481(24.7)	20.1-29.4	1		
Rainy	252/1357(18.6)	14.6-22.5	0.74	0.62-0.87	0.004
**Correct use of LLIN's**					
No	349/1305(26.7)	21.9-31.6	1		
Yes	269/1533(17.5)	13.8-21.3	0.74	0.62-0.87	0.005

The mean parasite density in positive asymptomatic children was 586 *P. falciparum *asexual forms per μL of blood (95%CI 504-680). According to multivariate random-effects linear regression model, increased parasite density was associated with some villages (Dokanmè and Satré) but not with age of children, season, ownership and use of LLINs (Table 4). *Plasmodium falciparum *gametocyte annual prevalence rate was 3.0% (95%CI 2.2-5.6) and differed significantly between the dry (3.8% (95% CI 2.9-4.8)) and the rainy season (2.2% (95%CI 1.4-3.0)), p = 0.008.

**Table 4:** Multivariate analysis of parasite density among positive asymptomatic children observed by cross-sectional surveys.

		Parasite density		
		
Variable	Number	Geometric average (95%CI)	Adjusted multiplicative factor (95%CI)	p-values
**Age (year)**				
<1	67	686(424-1109)	1	
1-2	205	734(541-994)	1.02(0.68-1.55)	0.900
3-5	346	497(422-587)	0.78(0.52-1.16)	0.175
**Sex**				
F	297	508(419-617)	1	
M	252	668(535-835)	1.29(1.01-1.63)	0.041
**Village**				
Aïdjèdo	59	398(272-581)	1	
Dokanmè	77	807(544-1197)	1.80 (1.10-2.95)	0.026
Kindjitokpa	99	509(388-668)	1.20(0.81-1.77)	0.302
Guézohoué	27	758(298-1928)	1.52(0.75-3.06)	0.195
Hékandji	82	489(344-695)	1.13(0.72-1.79)	0.528
Satré	129	732(538-995)	1.77(1.19-2.63)	0.013
Wanho	145	552(375-813)	1.37(0.88-2.14)	0.129
**Season**				
Dry	366	551(464-656)	1	
Rainy	252	640(510-803)	1.04(0.85-1.28)	0.657
**Correct use of LLIN's**				
No	256	684(552-848)	1	
Yes	362	525(435-633)	1.22(0.99-1.51)	0.057

### Clinical malaria observed by ACD

**Figure 2:**
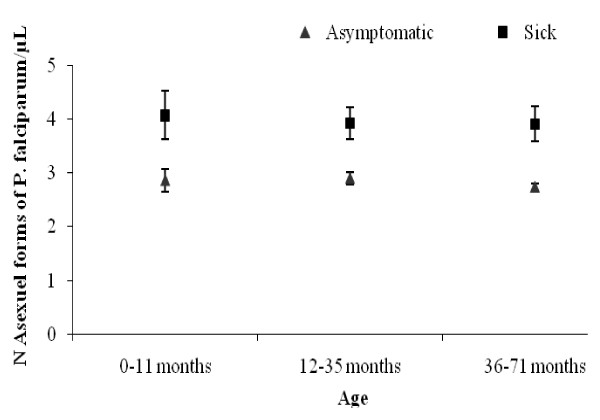
**Annual geometric mean density (95%CI) in parasite-positive asymptomatic (observed during CSS) and sick (found by ACD) children according to age**.

**Figure 3:**
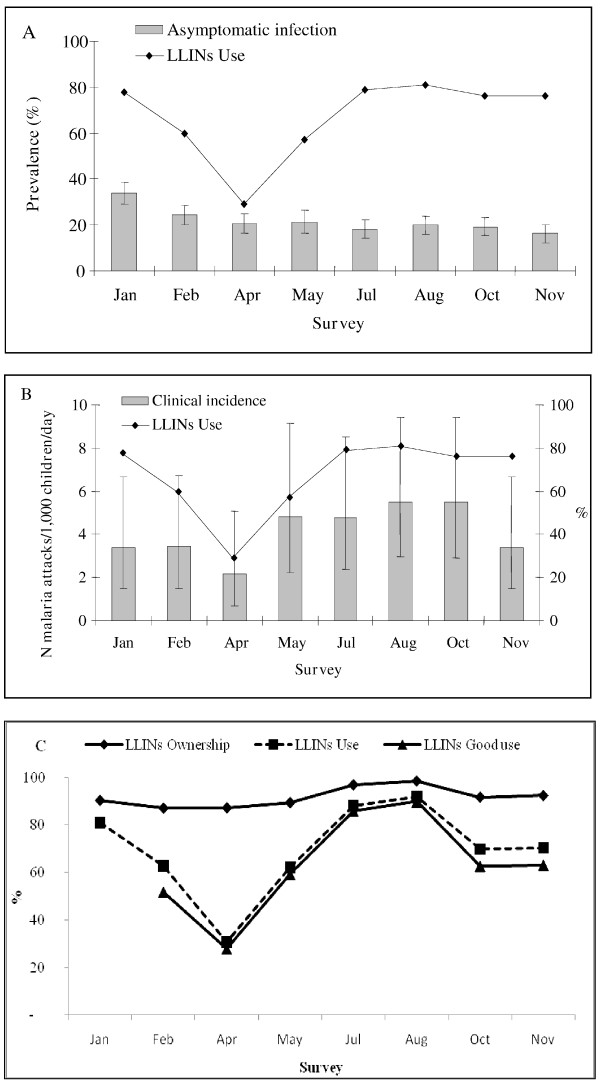
**Longitudinal study of malaria in seven villages of the health district of Ouidah-Kpomassè-Tori Bossito after the national distribution of LLINs in children aged 0-5 years**. **(A) **Asymptomatic infection: parasite prevalence rate with 95% confidence intervals and use of LLINs according to surveys. **(B) **Malaria disease: incidence rate of malaria attacks with 95% confidence intervals and use of treated nets according to surveys.**(C) **Ownership, Use and Correct use of LLINs according to surveys.

During the study 236 pathological episodes were detected. A total of 110 pathological episodes were parasite positive: 102 children with *P. falciparum *alone, three individuals with *P. malariae *alone, two individuals with *P. ovale *alone and three individuals with mixed infection. The *P. malariae *single infections showed densities of 480, 2,360 and 200 parasites/μL and *P. ovale *single infections showed densities of 4,800 and 9,800 parasites/μL. Three mixed infections with *P. falciparum *and *P. ovale *showed combined densities of 3,760 *Pf *+ 720 *Po*, 960 *Pf *+ 400 *Po *and 280 *Pf *+ 120 *Po *(Table 2). In all age groups, the mean parasite density was lower in healthy children than sick children (Figure 2). Four parasite positive cases with *P. falciparum *referred to the health centre were diagnosed as severe malaria with anaemia. Among the 236 pathological episodes, there were 74 episodes attributable to *P. falciparum *malaria (Table 5). The optimal pyrogenic parasite cut-off of 2,000 *P. falciparum *asexual blood forms per μL was determined as corresponding to levels of sensitivity and specificity of 94.0% and 94.5% respectively (Table 6). The mean annual clinical incidence rate was 1.5 per child per year (95%CI 1.2-1.9). Age and sex of children, season, village, the ownership, use and correct use of LLINs were not associated with the clinical malaria attacks in the Poisson regression model (Figure 3B, Table 7).

**Table 5:** Attributable fraction estimates of pathological episodes to falciparum malaria.

			Logistic model	
			
Trophozoites/μL	N asymptomatic children	N sick children	N clinical malaria cases	N other clinical cases (No malaria)
0	2220	131	0.0	131.0
1-99	112	5	0.3	4.7
100-249	84	7	0.9	6.1
250-499	87	5	0.9	4.1
500-999	94	5	1.4	3.6
1000-1499	62	1	0.3	0.7
1500-1999	28	4	1.7	2.3
2000-2999	44	3	1.5	1.5
3000-3499	12	3	1.7	1.3
3500-3999	8	3	1.8	1.2
4000-4999	18	2	1.3	0.7
5000-7499	23	5	3.5	1.5
7500-9999	16	3	2.4	0.6
10000-14999	13	5	4.3	0.7
≥15000	17	54	52.5	1.5
Total	2838	236	74.4	161.6

**Table 6:** Pyrogenic cut-off, sensitivity and specificity estimates by using attributable fraction (sensibility = number of malaria episodes ≥ cut-off/total of malaria episodes; specificity = number of no malaria episodes < cut-off/total of no malaria episodes).

Cut-off (trophozoites of *P. falciparum*/μL)	**N malaria episodes **≥ **pyrogenic cut-off**	N no malaria episodes < pyrogenic cut-off	Sensibility (%)	Specificity (%)
1	74.4	131.0	100.0	81.1
100	74.2	135.7	99.6	84.0
250	73.3	141.8	98.5	87.8
500	72.4	145.9	97.2	90.3
1000	71.0	149.6	95.4	92.6
1500	70.7	150.2	94.9	93.0

**2000**	**68.9**	**152.5**	**92.6**	**94.4**

3000	67.5	154.0	90.6	95.3
3500	65.8	155.3	88.4	96.2
4000	64.0	156.5	86.0	96.9
5000	62.7	157.2	84.2	97.3
7500	59.2	158.8	79.5	98.3
10000	56.8	159.4	76.3	98.6
> = 15000	52.5	160.1	70.5	99.1

**Table 7:** Multivariate regression analysis of malaria incidence taken into account the cumulative number of monitoring days

	Malaria episodes					
Variable	Person-day	N evocative malaria cases	N malaria cases*	Incidence per child per year (95%CI)	Adjusted Relative Risk (95%CI)	p-values
**Age (year)**						
<1	5542	77	18	1.19(0.77-1.83)	1	-
1-2	6034	84	30	1.81(1.28-2.58)	1.53(0.88-2.85)	0.104
3-5	6686	75	30	1.64(1.21-2.23)	1.38(0.94-2.45)	0.075
**Sex**						
F	9094	116	34	1.36(1.00-1.86)	1	-
M	9168	120	44	1.75(1.33-2.30)	1.27(0.86-1.88)	0.182
**Village**						
Aïdjèdo	2746	36	10	1.33(0.80-2.22)	1	
Dokanmè	2745	48	20	2.66(1.80-3.94)	1.89(0.98-3.65)	0.055
Kindjitokpa	2522	21	10	1.45(1.04-2.02)	1.04(0.56-1.94)	0.876
Guézohoué	2595	24	6	0.84(0.50-1.43)	0.60(0.29-1.25)	0.140
Hékandji	2536	38	7	1.01(0.55-1.84)	0.70(0.32-1.56)	0.320
Satré	2368	34	11	1.70(1.23-2.33)	1.21(0.66-2.23)	0.472
Wanho	2750	35	14	1.86(1.31-2.64)	1.34(0.70-2.57)	0.308
**Season**						
Dry	9355	92	36	1.40(1.00-1.97)	1	-
Rainy	8907	144	42	1.72(1.35-2.19)	1.22(0.78-1.91)	0.372
**Correct use of LLIN's**						
No	8280	92	28	1.23(0.87-1.75)	1	-
Yes	9982	144	50	1.83(1.44-2.32)	1.36(0.83-2.25)	0.181

*Parasite density ≥ 2000 asexual forms of *Plasmodium falciparum *per μL

### Entomological indexes

Overall 13,602 mosquitoes including 115 *An. gambiae sensu lato *(s.l.) (65 and 50 indoor and outdoor, respectively) and 67 *An. funestus *(40 and 27 indoor and outdoor, respectively) were caught in the seven villages. The number of CSP positive *An. gambiae s.l. *and *An. funestus *were nine and four respectively. The aggressiveness of culicidae and malaria vectors (*An. gambiae *s.l. and *An. funestus*) were 5,541 (95%CI 2,008-15,288) and 74 (95%CI 17-318) bites per human per year. The annual EIR was 5.3 (95%CI 1.1-25.9) infected bites per human per year. The 1014F kdr allele was present in both molecular M and S forms. The frequency of this mutation was respectively 0.47 (95%CI 0.37-0.57) and 0.61 (95%CI 0.43-0.80) in the M and S forms.

### Ownership and use of LLINs

The LLINs ownership rate reached 91.8% (2,769/3017; 95%IC 90.8-92.8) and remained high through the year. Use was significantly higher during the rainy season than the dry season 73% (1,062/1,451; 95%CI 70-75) and 67% (1,047/1,566; 95%CI 64-69) respectively, p = 0.0001. It significantly decreased to 31% (118/385; 95%CI 26-31) in the middle of dry season. Correct use was also the highest during the rainy season (68% (990/1,566; 95%CI 65-70)) compared to the dry season (42% (665/1,171; 95%CI 40-45)), p < 0.0001, (Figure 3C).

## Discussion

This prospective longitudinal study allowed the epidemiology of malaria in the health district of Ouidah-Kpomassè-Tori Bossito after the nation-wide distribution of LLINs to children in October 2007 to be characterized. Previous studies have described the epidemiology of malaria in Benin, by focusing mainly on malaria transmission [35] and on clinical and parasitological aspects as well in rural areas [36-38] as in the city of Cotonou [39]. Other authors [40] described the process indicators, results and impact of malaria control which were useful for the implementation of the monitoring and assessment system of ''Roll Back Malaria'' in Benin. The large scale and selective distribution of LLINs in Africa in the last decade were also the subject of several studies which concerned mainly the acceptability and/or the population perception without investigating their parasitological and clinical effects [41-47]. Pyrethroid resistance in malaria vectors has been observed in many African countries [7]. Nevertheless, no loss of effectiveness of LLINs has been reported at an operational level [48].

### Epidemiological description

The entomological findings showed that the health district of Ouidah-Kpomassè-Tori Bossito is a mesoendemic area with a mean annual EIR of 5.3 infected bites (95%CI 1.1-25.9). This EIR was found in conjunction with the annual prevalence rate of 21.8% (95%CI 19.1-24.4) observed in young asymptomatic children [29,49,50]. It confirms Velema's parasitological observations in the same area twenty years ago [36]. As regards the resistance of *An. gambiae *to pyrethroids, the L1014F *kdr *allele reached 50%, in accordance with previous studies carried out in southern Benin [8,18]. The annual infection rate increased with age in accordance with what is usually observed in mesoendemic area [50]. The high infection rate in the dry season could be influenced by the peak observed at the end of the rainy season (33%) just one month after the national distribution of LLINs (Figure 3A, Table 3). The parasite density of positive children did not vary with age group or season (Figure 2). These results may be attributed to the protection conferred by LLINs. In mesoendemic areas, the acquisition of immunity against malaria would develop gradually and bring about a decrease in parasitaemia with increasing age [51]. Here, where the level of parasite exposure was reduced by treated nets, immunity may be acquired more slowly [52,53]. The different rates of infection found in the villages can be explained by variety in their natural characteristics. The prevalence rate was the highest in Satré, Wanho, Kindjitopka and Hèkandji close to fresh water (Toho Lake), open water cisterns or swamps where breeding sites of anopheles are mostly found (Tables 1 and 3).

The calculated AF of pathological episodes to malaria helped to determine the optimum parasite pyrogenic cut-off at 2,000 *P. falciparum *asexual blood forms per μL. The use of AF to define the pyrogenic parasite cut-off allows the best trade-off between sensitivity and specificity level [54]. In stable malaria areas, *P. falciparum *parasitaemia is dependent on the season and age, which affects the malaria-AF of pathological episodes and thus the malaria case definition according to pyrogenic parasite density cut-off [33,55]. In the present study, the parasite density did not vary with season or age. Therefore, the AF could be considered the same whatever the season and the age group. The cut-off of 2,000 *falciparum *asexual blood forms per μL was close to the value of 1,000 found in mesoendemic area on children under 3 years [36] and to the 3,000 to 6,000 found in hyperendemic area among children aged 0 to 12 years in south of Benin respectively [37].

In the health district of Ouidah-Kpomassè-Tori Bossito, one pathological episode out of three was attributed to malaria. To avoid a maximum of missed cases the malaria case definition took into account signs evoking malaria or history of fever during the 48 hours preceding the first day of ACD as advised Mcguinness [33]. Mean annual incidence rate of falciparum clinical malaria was 1.5 per child per year. In *P. falciparum *high-endemic area, the pyrogenic cut-off of parasitaemia in persons of a given age is similar for all *Plasmodium *species [56]. Given the high parasite density, *P. malariae *could have been responsible for one malaria clinical case with 2,360 parasites/μL and *P. ovale *for two cases with a parasitaemia of (4,800 and 9,800 parasites/μL) respectively.

### Use of LLINs

In 2001 before the national distribution of LLINs, in south of Benin, 4.3% of household owned a treated net (ITN) and 2.4% of children under five years old slept under ITNs [40]. In 2006, ITNs possession was estimated to 25.6% and its utilization by the children less than 5 years was 21% in Ouidah [15]. After the national distribution of LLINs, ownership rose to over of 90% and was continuous over of the year (Figure 3C). Throughout the 12 months of the study, two children out of three were found sleeping under LLINs during unannounced and nocturnal inspections. Some studies have already concluded that free distribution of nets via a national campaign is effective in rapidly increasing their possession and use [42,57,58]. This high percentage of use may have been the result of adapted sensitization to the beliefs and behaviours of the communities and to the presence of medical staff assisted by a local village helper. Indeed, the success of sensitization depended strongly on the partnership between the study team and the local leaders as described by Paré Toé [47]. The 31% reduction of LLINs use during the dry season in Benin is comparable to that observed in most of the West African countries (Figure 3C) [24,41,43]. When populations were not bothered by the mosquitoes, they did not use the treated nets [47,59]. In the present longitudinal study, neither asymptomatic infection nor malaria attack was affected by the use of LLINs. The impact of LLINs was lower than expected since the correct use gave a 26% of individual protective effect only against infection without influencing malaria morbidity. Moreover, both curves of use and correct use of LLINs varied in the same way through the surveys (Figure 3C).

## Conclusion

In conclusion, the health district of Ouidah-Kpomassè-Tori Bossito is a mesoendemic area characterized by a moderate level of pyrethroid resistance of vectors and a high heterogeneity of malaria infection between villages. Malaria infection and disease did not vary through the year. The used LLINs rate was high and only the correct use of LLINs was found to reduce malaria infection without influencing malaria morbidity.

## Competing interests

The authors declare that they have no competing interests.

## Authors' contributions

The design of the study was conceived by FC and M-CH. Data were collected in the communities by GBD, AD and A-SBB. M-CH, AD and VC carried out laboratory analysis. CR, M-CH, GBD, AD and VC participated in the data analysis. The results were discussed by GBD, M-CH, AD, VC, CR, FC, MA, DK-G and AM. GBD and M-CH draft the manuscript. All authors read and approved the final manuscript.
